# Construction of a nomogram model for predicting the outcome of debulking surgery for ovarian cancer on the basis of clinical indicators

**DOI:** 10.3389/fonc.2024.1421247

**Published:** 2024-07-10

**Authors:** Yuanyuan Si, Ningjia Song, Yong Ji

**Affiliations:** ^1^ Department of Anesthesiology, Affiliated Hospital of Jiangnan University, Wuxi, China; ^2^ Wuxi Medical School, Jiangnan University, Wuxi, China

**Keywords:** ovarian cancer, nomogram, debulking surgery, ROC curve, calibration curve, clinical decision curve

## Abstract

**Objective:**

This study aimed to investigate the risk factors affecting satisfaction with debulking surgery for ovarian cancer and establish a preoperative clinical predictive model.

**Methods:**

Clinical data from 131 patients who underwent ovarian cancer debulking surgery at Jiangnan University Affiliated Hospital between 2016 and 2022 were collected. Patients were randomly separated into an experimental group and a control group in a 7:3 ratio. On the basis of intraoperative outcomes, patients were grouped as either surgery-satisfactory or surgery-unsatisfactory. Clinical indicators were compared through single-factor analysis between groups. Significantly different factors (*p* < 0.1) were further analyzed through multivariate logistic regression. A predictive nomogram model was developed and validated by receiver operating characteristic (ROC), calibration, and clinical decision curves.

**Results:**

Single-factor analysis revealed the significance of factors such as albumin levels, alkaline phosphatase (ALP), ECOG scores, CA125, HE4, and lymph node metastasis. Multivariate regression analysis identified albumin levels, ALP, ECOG scores, HE4, and lymph node metastasis as independent risk factors for satisfactory surgical outcomes in patients with ovarian cancer undergoing debulking surgery as (*p* < 0.05). A clinical predictive model was successfully constructed. ROC curves showed AUC values of 0.818 and 0.796 for the experimental and validation groups, respectively. Internal validation through the bootstrap method confirmed the model’s fit in both groups. Meanwhile, the clinical decision curve demonstrated the model’s high utility.

**Conclusion:**

Independent risk factors associated with satisfactory tumor reduction in patients with ovarian cancer undergoing debulking surgery included decreased albumin levels, ALP > 137 U/L, ECOG = 1 score, HE4 > 140 pmol/L, and lymph node metastasis. Constructing a clinical predictive model through logistic regression analysis enables individualized testing and maximizes clinical benefits.

## 1 Introduction

Epithelial ovarian cancer (EOC) often manifests subtly without specific early symptoms, and it ranks the highest in terms of fatality rates among female reproductive system malignancies (1, 2). Standard treatment for advanced EOC involves initial debulking surgery followed by postoperative platinum-based chemotherapy. For challenging cases wherein satisfactory tumor reduction is difficult to achieve, a strategy comprising neoadjuvant chemotherapy prior to interval debulking surgery may be considered (2–5). Notably, complete cytoreduction without visible residual disease postsurgery serves as an independent prognostic factor influencing patient outcomes (6–8). Therefore, predicting successful tumor reduction preoperatively is crucial to guiding the initial treatment plan in clinical settings. Tumor markers, hematologic indicators, radiological images, genetic analysis by microarrays, and diagnostic laparoscopy have been utilized in several studies to predict the outcomes of debulking surgery (9–15). The use of surgery instruments, access methods, protective stoma, and intraabdominal drainage have notable effects (16–18). Nevertheless, the effectiveness of these parameters has proven unsatisfactory and constrained in accuracy. This retrospective study analyzed patients with ovarian cancer undergoing debulking surgery at our institution from January 2016 to December 2022 to identify key factors associated with achieving effective tumor reduction.

## 2 Material and methods

### 2.1 Clinical data

This retrospective study included 131 patients with ovarian cancer who underwent debulking surgery at the Jiangnan University Affiliated Hospital from January 2016 to December 2022. The dataset was divided randomly into an experimental group (91 cases) and a validation group (40 cases) in a 7:3 ratio. Comparative analysis revealed no statistically significant differences between the two groups (*p* > 0.05).

Inclusion criteria were as follows: (1) pathologically confirmed ovarian cancer; (2) receipt of debulking surgery for ovarian cancer; and (3) availability of complete clinical data.

Exclusion criteria were as follows: (1) individuals with severe cardiovascular diseases; (2) prior exposure to chemotherapy, radiotherapy, or immunotherapy; and (3) patients with mental illnesses.

### 2.2 Data collection

Clinical information was extracted from electronic medical records. It included age, height, weight, menopausal status, hypertension history, ascites presence, reproductive history, blood parameters, blood glucose levels, ECOG scores, liver function tests, tumor markers (CEA, CA125, HE4, and CA199), interventions, surgical methods, FIGO stage, pathological types, and lymph node status.

### 2.3 Assessment criteria

The Gynecologic Oncology Group classifies postoperative residual tumors as R0 (no macroscopic residual tumor); R1 (residual tumor size 0.1–1 cm), which is considered as satisfactory surgery; and R2 (residual tumor size 1.1–2 cm), which is deemed as unsatisfactory surgery.

### 2.4 Statistical analysis

Statistical analyses were performed by SPSS 27.0 software. The continuous normality and homogeneity of variance data were assessed. Normally distributed data were depicted as mean ± standard deviation. Independent sample *t*-test was used for intergroup comparisons. Categorical data were presented as frequencies and percentages and compared via the Chi-square test. Risk factors for surgical satisfaction in patients with ovarian cancer were analyzed by multivariable logistic regression. Model accuracy was evaluated through the Hosmer–Lemeshow test, and receiver operating characteristic (ROC) curves were plotted. Predictive model visualizations were created using the R software packages rms and rmda.

## 3 Results

### 3.1 Comparison of clinical data between experimental and validation groups

The analysis of patients’ clinical data in the experimental and validation groups revealed no statistically significant differences (*p* > 0.05) (**Table 1**).

**Table 1:** Comparison of clinical data between the experimental group and the validation group.

Variables	Experimental (*n* = 91)	Validation (*n* = 40)	*χ* ^2^/*t*	*p*-value
Age(years)	59.24±10.60	59.25±9.37	0.00	1.00
BMI	25.37±3.33	24.37±3.37	1.57	0.12
Menopausal[n(%)]			0.41	0.52
no	12(13.2)	3(7.5)		
yes	79(86.8)	37(92.5)		
hypertension [n(%)]			2.61	0.11
no	57(62.6)	19(47.5)		
yes	34(37.4)	21(52.5)		
Ascites[n(%)]			2.13	0.14
no	42(46.2)	24(60.0)		
yes	49(53.8)	16(40.0)		
Fertility[n(%)]			0.57	0.45
≤2times	83(91.2)	34(85.0)		
>2times	8(8.8)	6(15.0)		
NLR	3.59±2.84	2.97±1.65	1.28	0.2
Albumin(g/L)	38.69±5.51	38.66±8.15	0.03	0.98
ALP[n(%)]			1.31	0.25
≤135U/L	83(91.2)	33(82.5)		
>135U/L	8(8.8)	7(17.5)		
Glu	5.15±0.74	5.37±1.29	-1.21	0.23
ECOG[n(%)]			2.33	0.13
0	80(87.9)	31(77.5)		
1	11(12.1)	9(22.5)		
CEA(ng/ml)	3.25±10.74	1.56±0.87	0.99	0.32
CA125[n(%)]			0.39	0.82
≤500U/ml	34(37.4)	16(40.0)		
≤1000U/ml	27(29.7)	13(32.5)		
>1000U/ml	30(33)	11(27.5)		
HE4[n(%)]			0.02	0.89
<140 pmol/L	58(63.7)	25(62.5)		
≥140 pmol/L	33(36.3)	15(37.5)		
CA199[n(%)]			0.34	0.56
<37U/mL	71(78.0)	33(82.5)		
≥37U/mL	20(22.0)	7(17.5)		
Interventions [n(%)]			0.11	0.74
PDS	61(67.0)	28(70.0)		
IDS	30(33.0)	12(30.0)		
Operation [n(%)]			0.04	0.85
Laparoscopy	85(93.4)	37(92.5)		
Laparotomy	6(6.6)	3(7.5)		
FIGO stage[n(%)]			0.02	0.89
I~II	15(16.5)	7(17.5)		
III~IV	76(83.5)	33(82.5)		
pathology[n(%)]			4.27	0.13
Serous	66(72.5)	34(85.0)		
Mucinous	5(5.5)	3(7.5)		
others	20(22.0)	3(7.5)		
Lymph node metastasis [n(%)]			1.43	0.23
no	42(46.2)	23(57.5)		
yes	49(53.8)	17(42.5)		

The comparison among groups was statistically significant,P<0.05

NLR, neutrophil-to-lymphocyte ratio; ALP, alkaline phosphatase; Glu, glucose.

### 3.2 Development of the clinical predictive model

#### 3.2.1 Comparison of clinical data within the experimental group

Significant differences (*p* < 0.1) were observed in the comparison of clinical parameters between patients with ovarian cancer in the satisfactory and unsatisfactory surgical groups. Parameters showing variance included albumin and alkaline phosphatase (ALP) levels, ECOG score, CA125, HE4, FIGO staging, and lymph node metastasis. Conversely, no statistically significant differences (*p* > 0.1) were found in age, BMI, menopausal status, hypertension and ascites history, reproductive history, NLR, blood glucose levels, CEA, CA199, interventions received, surgical methods, FIGO stage, and pathological types (**Table 2**).

**Table 2:** Comparison of clinical data between surgical satisfaction group and surgical dissatisfaction group.

Variables	Satisfaction (*n* = 71)	Dissatisfaction (*n* = 20)	*χ* ^2^ */t*	*p*-value
Age(years)	59.14±10.32	59.6±11.82	-0.17	0.87
BMI	25.14±3.46	26.18±2.73	-1.24	0.22
Menopausal[n(%)]			0.12	0.73
no	9(12.7)	3(15.0)		
yes	62(87.3)	17(85.0)		
hypertension [n(%)]			0.06	0.81
no	44(62.0)	13(65.0)		
yes	27(38.0)	7(35.0)		
Ascites[n(%)]			2.69	0.1
no	36(50.7)	6(30.0)		
yes	35(49.3)	14(70.0)		
Fertility situation [n(%)]			0.29	0.59
≤2times	64(90.1)	19(95.0)		
>2times	7(9.9)	1(5.0)		
NLR	3.29±1.74	4.64±5.07	-1.17	0.26
Albumin(g/L)	39.2±5.48	36.9±5.36	1.66	0.09
ALP[n(%)]			4.09	0.04
≤135U/L	67 (94.4)	16 (80.0)		
>135U/L	4 (5.6)	4 (20.0)		
Glu	5.14±0.78	5.2±0.63	-0.33	0.75
ECOG[n(%)]			5.68	0.02
socore =0	65(91.5)	15(75)		
socore =1	6(8.5)	5(25)		
CEA(ng/ml)	3.76±12.11	1.46±1.18	0.84	0.4
CA125[n(%)]			9.84	0.01
≤500U/ml	27 (38.0)	7 (35.0)		
≤1000U/ml	17 (23.9)	10 (50.0)		
>1000U/ml	27 (38.0)	3 (15.0)		
HE4[n(%)]			3.69	0.04
<140 pmol/L	49 (69.0)	9 (45.0)		
≥140 pmol/L	22(31.0)	11(55.0)		
CA199[n(%)]			0.12	0.73
<37U/mL	54 (77.1)	16 (80.0)		
≥37U/mL	16 (22.9)	4 (20.0)		
Interventions [n(%)]			0.08	0.78
PDS	48(67.6)	13(65.0)		
IDS	23(32.4)	7(35.0)		
Operation [n(%)]			0.18	0.69
Laparoscopy	66(93.0)	19(95.0)		
Laparotomy	5(7.0)	1(5.0)		
FIGO stage[n(%)]			1.22	0.27
I~II	13(18.3)	1(5.0)		
III~IV	58(81.7)	19(95.0)		
pathology[n(%)]			4.29	0.11
Serous	49(69.1)	17(85.0)		
Mucinous	5(7.0)	0(0.0)		
others	17(23.9)	3(15.0)		
Lymph node metastasis [n(%)]			4.616	0.03
no	37(52.1)	5(25.0)		
yes	34(47.9)	15(75.0)		

The comparison among groups was statistically significant,P<0.05

NLR, neutrophil-to-lymphocyte ratio; ALP, alkaline phosphatase; Glu,glucose.

#### 3.2.2 Establishment of the clinical predictive model

Variables with *p* < 0.1 from **Table 2** were utilized as independent factors in the multivariable logistic regression model, with surgical satisfaction as the dependent variable. Albumin, ALP, ECOG scores, HE4, and lymph node metastasis were identified as key independent risk factors (*p* < 0.05) affecting the satisfaction level of patients with ovarian cancer undergoing debulking surgery. The model equation was as follows: 6.562 − 0.192 × albumin − 2.179 × ALP + 2.027 × ECOG score + 1.536 × HE4 + 1.740 × lymph node metastasis (**Table 3**).

**Table 3:** Multivariate logistic regression analysis of risk factors affecting surgical satisfaction.

Variables	*β*	SE	Wald	*p*-value	OR	95% CI	
						Lower	Upper
Albumin	− 0.15	0.07	4.48	0.03	0.86	0.75	0.99
ALP	− 2.02	0.90	5.11	0.02	0.13	0.02	0.76
ECOG	1.75	0.85	4.24	0.04	5.77	1.09	30.57
HE4	− 1.31	0.66	3.98	0.04	0.27	0.08	0.98
Lymph node metastasis	− 1.40	0.69	4.12	0.04	0.25	0.06	0.95

ALP, alkaline phosphatase.

#### 3.2.3 Nomogram model

**Figure 1:**
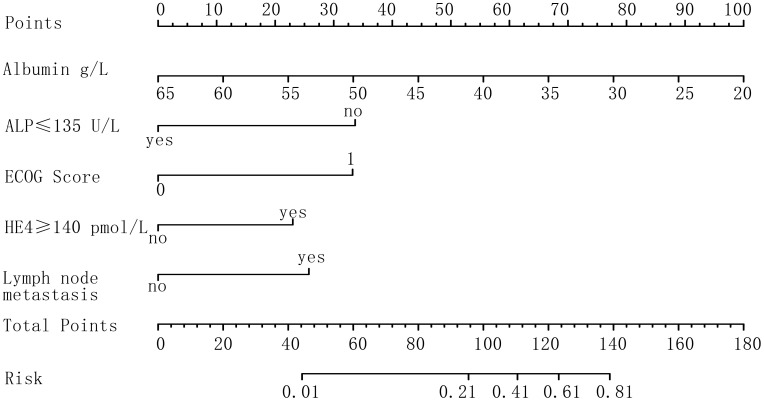
Nomogram of risk factors for debulking surgery in patients with EOC.

On the basis of the results of multivariable logistic regression analysis, a nomogram model was generated using the R package rms. Notably, a decrease in albumin levels by five units resulted in an 11-point increase in the model score. Compared with ALP levels at or below 135 U/L, ALP > 135 U/L contributed to a 33-point increase in the model score. Additionally, an ECOG score of 1 versus 0 led to a 32-point increase in the model score. HE4 > 140 pmol/L contributed to a 22-point score increase, whereas the presence of lymph node metastasis added 25 points to the model score (**Figure 1**).

### 3.3 Validation of the nomogram model

#### 3.3.1 ROC curve

**Figure 2:**
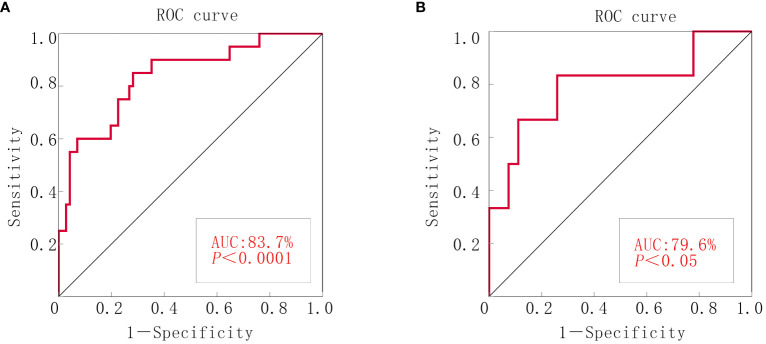
ROC curve of the nomograms for debulking surgery. **(A)** The experimental group. **(B)** The validation group.

ROC curves were generated by SPSS software to assess model accuracy. The AUC and 95% CI were calculated. The results revealed an AUC of 0.818 (*p* < 0.000; 95% CI: 0.721–0.915) for the experimental group and 0.796 (*p* < 0.05; 95% CI: 0.566–1.000) for the validation group. These findings suggested the effective prediction of postsurgery satisfaction levels in patients with ovarian cancer (**Figure 2**).

#### 3.3.2 Calibration curve

**Figure 3:**
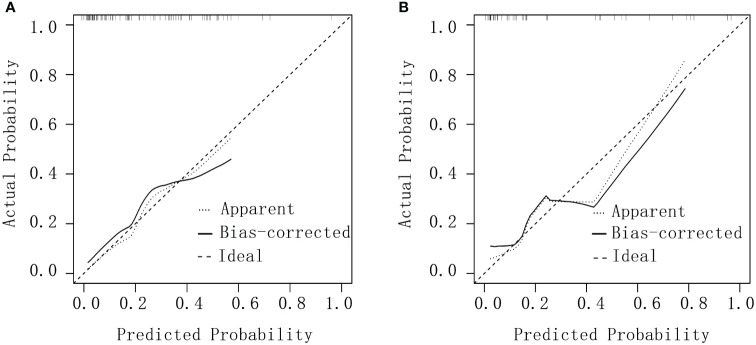
Calibration curves for debulking surgery. **(A)** The experimental group. **(B)** The validation group.

Calibration curves were plotted for both groups by using the rms package in R software and assessed via a Hosmer–Lemeshow goodness-of-fit test. Excellent consistency between actual and predicted probabilities was observed, indicating high predictive accuracy (**Figure 3**).

#### 3.3.3 Clinical decision curve

**Figure 4:**
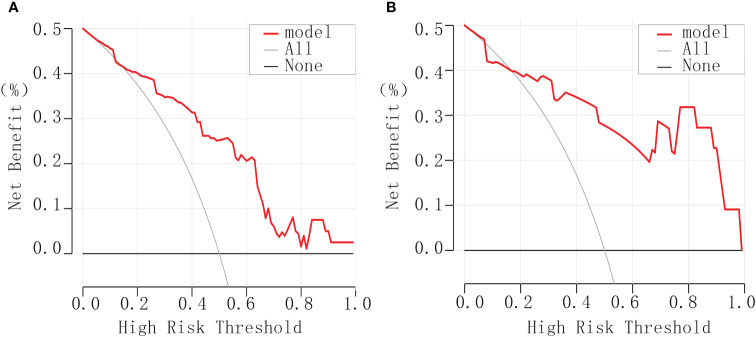
Clinical decision curves for debulking surgery. **(A)** The experimental group. **(B)** The validation group.

The clinical decision curve generated by utilizing the rmda package in R software illustrated the model’s performance relative to reference lines. Notably, it demonstrated the model’s high safety levels, substantial net benefits, and considerable clinical value (**Figure 4**).

## 4 Discussion

Among malignancies in the female reproductive system, EOC ranks as the third most prevalent and lethal form. In 2020, China reported 55,342 new cases of ovarian cancer, with a mortality rate of 67.8% (19). Initial treatment protocols for ovarian cancer are determined after evaluation by gynecologic oncologists. When optimal debulking is not possible, neoadjuvant chemotherapy rather than immediate blind debulking surgery may precede surgical intervention posttumor reduction. Research indicated a direct correlation between the extent of cytoreduction during ovarian cancer surgery and the survival rates of patients, with a 25% increase in patient survival observed for every additional 10% resection achieved (20).

Elevated serum levels of the tumor marker HE4 have been linked to a high likelihood of unsatisfactory results in debulking surgeries for EOC (21, 22). The independent predictive power of HE4 for surgical outcomes remains a topic of debate and lacks a unanimous optimal threshold value (**Supplementary Table S1**). In this study, the group experiencing unsatisfactory outcomes exhibited notably higher HE4 levels than their counterparts with satisfactory outcomes. Multifactorial analysis identified HE4 as an independent risk factor affecting the success of debulking surgeries, with a critical threshold of 140 pmol/L. Furthermore, the presence of lymph node metastasis has been shown to influence surgical outcomes, particularly increasing the likelihood of unsatisfactory results in patients with ovarian cancer and positive lymph node involvement (23).

Moreover, research has highlighted the remarkable association of albumin levels, serum ALP, and ECOG score with the outcomes of debulking surgery for epithelial ovarian cancer.

Previous large-scale retrospective studies demonstrated that serum albumin levels are a significant independent prognostic marker for patients with ovarian cancer undergoing debulking surgery (24, 25). Studies have linked hypoalbuminemia (albumin < 35 g/L) to increased postoperative complications and mortality rates in epithelial ovarian cancer (26–28). Notably, low albumin levels are correlated with a high likelihood of residual lesions postsurgery (27). The present study showed that decreasing albumin levels correspond to an increased probability of unsatisfactory outcomes in debulking surgery for ovarian cancer. Elevated serum ALP levels are essential diagnostic indicators for skeletal issues and exhibit a notable correlation with bone metastasis in breast cancer (29). They are associated with unfavorable prognostic outcomes, indicating a risk factor for unsatisfactory debulking surgery results in breast cancer. The data in this study suggested that an advantageous outcome of debulking surgery may be obtained if ALP can be normalized below 135 U/L. The ECOG score serves as a measure of patient physical capacity, reflecting overall health status and treatment tolerance (30). Low ECOG scores indicate good treatment endurance and are linked to prolonged patient survival. ECOG scores were identified as an independent risk factor for satisfactory debulking surgery outcomes.

In summary, factors such as HE4, lymph node metastasis, albumin, ALP, and ECOG scores play pivotal roles in influencing the success of debulking surgery for epithelial ovarian cancer. The developed line plot model demonstrated substantial predictive value for identifying unsatisfactory debulking surgery outcomes. Given the limited case selection in this study, potential biases may exist in the data. Therefore, future studies should involve large sample sizes to validate the present findings and enhance the accuracy of our predictive model.

## Author contributions

YS: Investigation, Writing – original draft. NS: Investigation, Methodology, Resources, Validation, Writing – review & editing. YJ: Data curation, Validation, Writing – review & editing.

## Supplementary material

Supplementary Table 1 Studies were selected by exploring the PubMed database with a combination of the keywords “ovarian cancer”, “debulking surgery” and “HE4”. We took into consideration studies published after the year 2000 and the results are summarized in **Supplementary Table S1**.
