# Supplementary material for: Construction of a nomogram model for predicting the outcome of debulking surgery for ovarian cancer on the basis of clinical indicators
Source: Front Oncol. 2024 Jul 10;14:1421247. doi: 10.3389/fonc.2024.1421247 (PMC11266020; doi:10.3389/fonc.2024.1421247)
Supplement: Supplementary Table 1 — Studies were selected by exploring the PubMed database with a combination of the keywords “ovarian cancer”, “debulking surgery” and “HE4”. We took into consideration studies published after the year 2000 and the results are summarized in Supplementary Table S1 . [file Table_1.docx]

| **The results of the literature review are summarized in Table S1** | | | | | | | | |
| --- | --- | --- | --- | --- | --- | --- | --- | --- |
| First Author | Publication Years | Patients（No.） | Cut off point (pmol/L) | prediction model for debulking surgery | | | | |
|  |  |  |  | Sensitivity | Specificity | PPV | NPV | AUC |
| Amal AlSomairi[1] | 2024 | 123 | 65.5 | 69.10% | 41.70% |  |  | 61.90% |
| Yue Jia[2] | 2024 | 76 | 431.55 | Independent predictor by univariate analysis (OR=4.356) | | | | |
| Min Li[3] | 2022 | 115 | 717 | Independent predictor by multivariate analysis | | | | |
| Daniela Furrer[4] | 2019 | 313 | 277 | 75% | 49% |  |  | 64.20% |
| Manikandan Lakshmanan[5] | 2019 | 149 | Postoperative/Preoperative＜80% |  |  | 95.60% | 74.10% |  |
| Tuulia Vallius[6] | 2017 | 49 | nadir HE4 value | Independent predictor by multivariate analysis | | | | |
| Francesco Plotti[7] | 2017 | 114 | 226 | 75% | 85% | 87% | 70% |  |
| Vesna Paunovic[8] | 2017 | 50 | 413 | odds ratio 4.921 | | | | |
| Ying Shen[9] | 2016 | 82 | 353.22 | 77.40% | 75% |  |  | 75.80% |
|  |  |  | 154.3 | 93.20% | 69% |  |  | 79.30% |
| Zhijian Tang[10] | 2015 | 90 | 473 | 81% | 56% | 67% | 73% |  |
| Anita Monika Chudecka-Glaz[11] | 2014 | 56 | 218.43 | 86.60% | 91.30% | 92.90% | 84% |  |
| Tuulia Vallius[12] | 2014 | 25 | 389 | 67% | 100% |  |  | 94.70% |
| Elena Ioana Braicu[13] | 2014 | 73 | 72 | 88.10% | 47.90% |  |  |  |
|  |  |  | 250 | 52% | 93.80% |  |  |  |
| Zhijun Yang[14] | 2013 | 180 | 600 | 77% | 32% |  |  |  |
| Jiheum Paek[15] | 2011 | 78 | 155 | Independent predictor by multivariate analysis | | | | |

PPV , positive predictive value ; NPV , negative predictive value ; AUC ,area under curve ;

**Reference:**

1. AlSomairi A, Himayda S, Altelmesani A, Lee YJ, Lee JY: **Prognostic value of HE4 in advanced-stage, high-grade serous ovarian cancer: Analysis of HE4 kinetics during NACT, predicting surgical outcome and recurrence in comparison to CA125**. *Gynecol Oncol* 2024, **181**:155-161.

2. Jia Y, Jiang Y, Fan X, Zhang Y, Li K, Wang H, Ning X, Yang X: **Preoperative serum level of CA153 and a new model to predict the sub-optimal primary debulking surgery in patients with advanced epithelial ovarian cancer**. *World J Surg Oncol* 2024, **22**(1):64.

3. Li M, Zhang T, Zhu J, Li Y, Chen W, Xie Y, Zhang W, Chen R, Wei W, Wang G *et al*: **Risk factors of perioperative complications and management with enhanced recovery after primary surgery in women with epithelial ovarian carcinoma in a single center**. *Oncol Lett* 2022, **23**(5):155.

4. Furrer D, Grégoire J, Turcotte S, Plante M, Bachvarov D, Trudel D, Têtu B, Douville P, Bairati I: **Performance of preoperative plasma tumor markers HE4 and CA125 in predicting ovarian cancer mortality in women with epithelial ovarian cancer**. *PLoS One* 2019, **14**(6):e0218621.

5. Lakshmanan M, Kumar V, Chaturvedi A, Misra S, Gupta S, Akhtar N, Rajan S, Jain K, Garg S: **Role of serum HE4 as a prognostic marker in carcinoma of the ovary**. *Indian J Cancer* 2019, **56**(3):216-221.

6. Vallius T, Hynninen J, Auranen A, Matomäki J, Oksa S, Roering P, Grènman S: **Postoperative human epididymis protein 4 predicts primary therapy outcome in advanced epithelial ovarian cancer**. *Tumour Biol* 2017, **39**(2):1010428317691189.

7. Plotti F, Scaletta G, Capriglione S, Montera R, Luvero D, Lopez S, Gatti A, De Cicco Nardone C, Terranova C, Angioli R: **The Role of HE4, a Novel Biomarker, in Predicting Optimal Cytoreduction After Neoadjuvant Chemotherapy in Advanced Ovarian Cancer**. *Int J Gynecol Cancer* 2017, **27**(4):696-702.

8. Paunovic V, Protrka Z, Ardalic D, Paunovic T: **Usefulness of human epididymis protein 4 in predicting optimal cytoreductive therapy in patients with advanced ovarian cancer**. *J buon* 2017, **22**(1):29-33.

9. Shen Y, Li L: **Serum HE4 superior to CA125 in predicting poorer surgical outcome of epithelial ovarian cancer**. *Tumour Biol* 2016, **37**(11):14765-14772.

10. Tang Z, Chang X, Ye X, Li Y, Cheng H, Cui H: **Usefulness of human epididymis protein 4 in predicting cytoreductive surgical outcomes for advanced ovarian tubal and peritoneal carcinoma**. *Chin J Cancer Res* 2015, **27**(3):309-317.

11. Chudecka-Głaz AM, Cymbaluk-Płoska AA, Menkiszak JL, Sompolska-Rzechuła AM, Tołoczko-Grabarek AI, Rzepka-Górska IA: **Serum HE4, CA125, YKL-40, bcl-2, cathepsin-L and prediction optimal debulking surgery, response to chemotherapy in ovarian cancer**. *J Ovarian Res* 2014, **7**:62.

12. Vallius T, Hynninen J, Auranen A, Carpén O, Matomäki J, Oksa S, Virtanen J, Grénman S: **Serum HE4 and CA125 as predictors of response and outcome during neoadjuvant chemotherapy of advanced high-grade serous ovarian cancer**. *Tumour Biol* 2014, **35**(12):12389-12395.

13. Braicu EI, Chekerov R, Richter R, Pop C, Nassir M, Loefgren H, Stamatian F, Muallem MZ, Hall C, Fotopoulou C *et al*: **HE4 expression in plasma correlates with surgical outcome and overall survival in patients with first ovarian cancer relapse**. *Ann Surg Oncol* 2014, **21**(3):955-962.

14. Yang Z, Luo Z, Zhao B, Zhang W, Zhang J, Li Z, Li L: **Diagnosis and preoperative predictive value of serum HE4 concentrations for optimal debulking in epithelial ovarian cancer**. *Oncol Lett* 2013, **6**(1):28-34.

15. Paek J, Lee SH, Yim GW, Lee M, Kim YJ, Nam EJ, Kim SW, Kim YT: **Prognostic significance of human epididymis protein 4 in epithelial ovarian cancer**. *Eur J Obstet Gynecol Reprod Biol* 2011, **158**(2):338-342.
